# Granulomatosis With Polyangiitis Manifesting With Diabetes Insipidus and Cranial Nerve Involvement: A Case Report and Review of the Literature

**DOI:** 10.1002/ccr3.71429

**Published:** 2025-11-05

**Authors:** Mina Kiafar, Goharsharieh Alishiri, Ahmadreza Sadeghi, Amirreza Paksaz, Leila Khezrloo, Soheila Borji

**Affiliations:** ^1^ Department of Internal Medicine, Faculty of Medicine Alborz University of Medical Sciences Karaj Iran; ^2^ Students Research Committee, Faculty of School of Medicine, Ardabil University of Medical Sciences Ardabil Iran; ^3^ Student Research Committee Alborz University of Medical Sciences Karaj Iran

**Keywords:** case report, cranial nerve, diabetes insipidus, granulomatosis with polyangiitis, hypophysitis, oculomotor nerve, pituitary gland, vasculitis

## Abstract

This report describes a rare case of granulomatosis with polyangiitis presenting with diabetes insipidus and third cranial nerve involvement. Given the wide range of vasculitis presentations, this case highlights the importance of considering such diagnoses in patients with diverse signs and symptoms to prevent misdiagnosis or delayed diagnosis and to utilize targeted laboratory and paraclinical studies based on clinical suspicion to achieve an accurate diagnosis.

## 1 Introduction

Granulomatosis with polyangiitis (GPA) is a form of antineutrophil cytoplasmic antibody (ANCA)‐associated vasculitis that is characterized by necrotizing small‐vessel vasculitis and mostly affects the upper respiratory tract, lungs, and kidneys. Among these, the nasal cavity and paranasal sinuses are the most common sites of involvement and may be the initial clinical manifestations [1, 2]. However, GPA can potentially involve almost any organ, such as the central nervous system, of which pituitary gland involvement is a rare presentation and occurs in less than 1% of cases [3, 4]. Hypophysitis is a general term that includes a broad range of inflammatory disorders affecting the pituitary gland. Patients with hypophysitis mostly present with headaches along with anterior and/or posterior pituitary dysfunction based on the affected area [5]. Among the autoimmune etiologies causing hypophysitis, GPA is an uncommon systemic disease of unknown etiology that affects both sexes and a wide age range. The diagnosis of GPA is based on clinical suspicion of systemic vasculitis, serologic ANCA testing, and histological evidence from tissue biopsy. Additionally, a structured evaluation of all the organs possibly involved is necessary [1, 6]. Treatment of GPA relies mainly on various immunosuppressing agents for remission induction and maintenance [7]. Treatment for GPA results in remission most of the time, but in the case of pituitary gland involvement, recovery of pituitary function is rare [1, 8]. Although there are few reports investigating diabetes insipidus in GPA, here, we present the first reported case of GPA manifested with central diabetes insipidus along with third cranial nerve involvement. A comprehensive PubMed search revealed only one previous report of third cranial nerve involvement following GPA; however, that case was due to meningocerebral inflammation, a general involvement in the central nervous system with other neurological manifestations. Our case is the first reported instance of isolated third cranial nerve involvement associated with pituitary involvement due to GPA [9].

## 2 Case History/Examination

**FIGURE 1:**
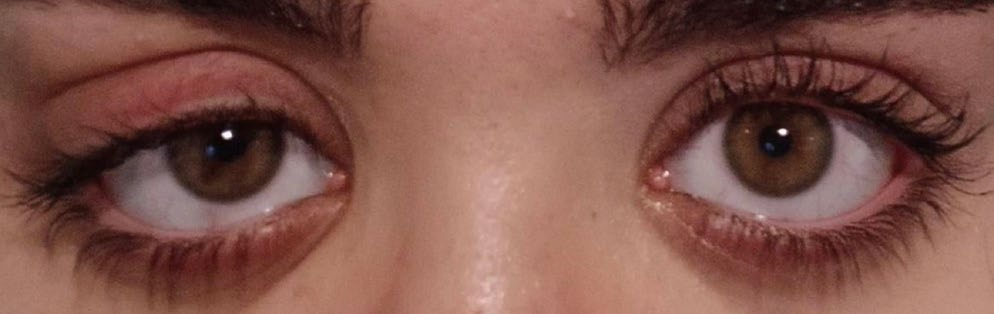
Unilateral ptosis and mydriasis of the patient's right eye.

A 21‐year‐old woman was referred to our hospital by an endocrinologist for further evaluation of diabetes insipidus. She had a history of polydipsia and polyuria approximately 3 months prior, for which diabetes insipidus was diagnosed and treated with desmopressin, to which she had a partial response, and her urinary symptoms, such as frequency, polyuria, and nocturia, had not completely disappeared. She also presented headaches and dyspnea at the time of admission to our hospital. She experienced headaches approximately four times per week beginning 6 months prior, which were nonpulsatile, non‐positional, localized at the frontal region with pressure feeling, photophobia, phonophobia, and response to painkillers. In addition to headache, the patient mentioned intermittent blurred vision and vertical diplopia, specifically at the time of headache that continued for a maximum of 2 h, with no evidence of fatigability. Her dyspnea, which was not accompanied by cough or hemoptysis, started approximately a few months prior in the winter and worsened over time. She had a history of the same condition every year at the same time, which was attributed to seasonal allergies. Further history‐taking and reviewing of systems revealed other complaints, such as fatigue, weight loss, and a history of epistaxis and gum bleeding, which occur occasionally. She also mentioned hearing loss and tinnitus, which are not evaluated. In her past medical history, in addition to diabetes insipidus, she had recurrent sinusitis for 2 years, which was followed by two episodes of surgical treatment last year without a desirable therapeutic effect. There was no other medication in the drug history except desmopressin nasal spray, and no history of drug addiction or smoking was given. The patient's family history was nonspecific, except for her grandmother, who was a known case of rheumatoid arthritis. In the initial evaluation, the patient was conscious, and there was no evidence of respiratory distress. She had a blood pressure of 113/78 mmHg, a pulse rate of 81/min, a respiratory rate of 16/min, and an SpO2 of 98%. On physical examination, a saddle nose deformity was observed, and further evaluation revealed a perforated nasal septum. Additionally, unilateral ptosis and mydriasis of the right eye (Figure 1), which suggest third cranial nerve compression, were present. Other physical examinations, such as heart and lung auscultation, were unremarkable. Finally, the patient was admitted to the internal ward and underwent several laboratory tests and paraclinic studies during hospitalization.

## 3 Differential Diagnosis, Investigations, and Treatment

**FIGURE 2:**
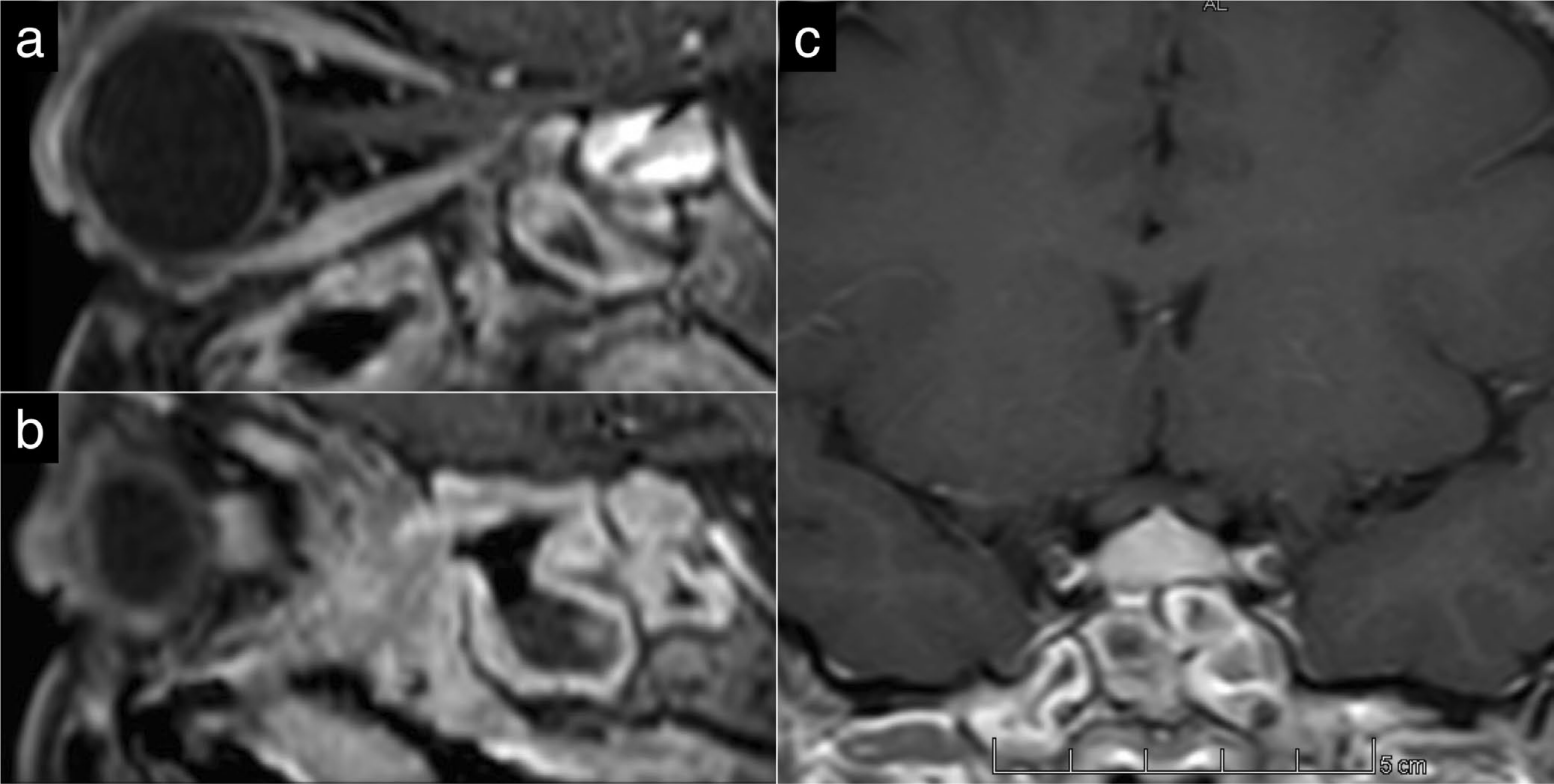
Brain MRI. (a) Enhancement of the pituitary stalk in favor of hypophysitis (sagittal plane); (b) Mucosal thickening of sphenoid sinus in favor of sinusitis; (c) Enhancement of the pituitary stalk and mucosal thickening of the sphenoid sinus (coronal plane).

**FIGURE 3:**
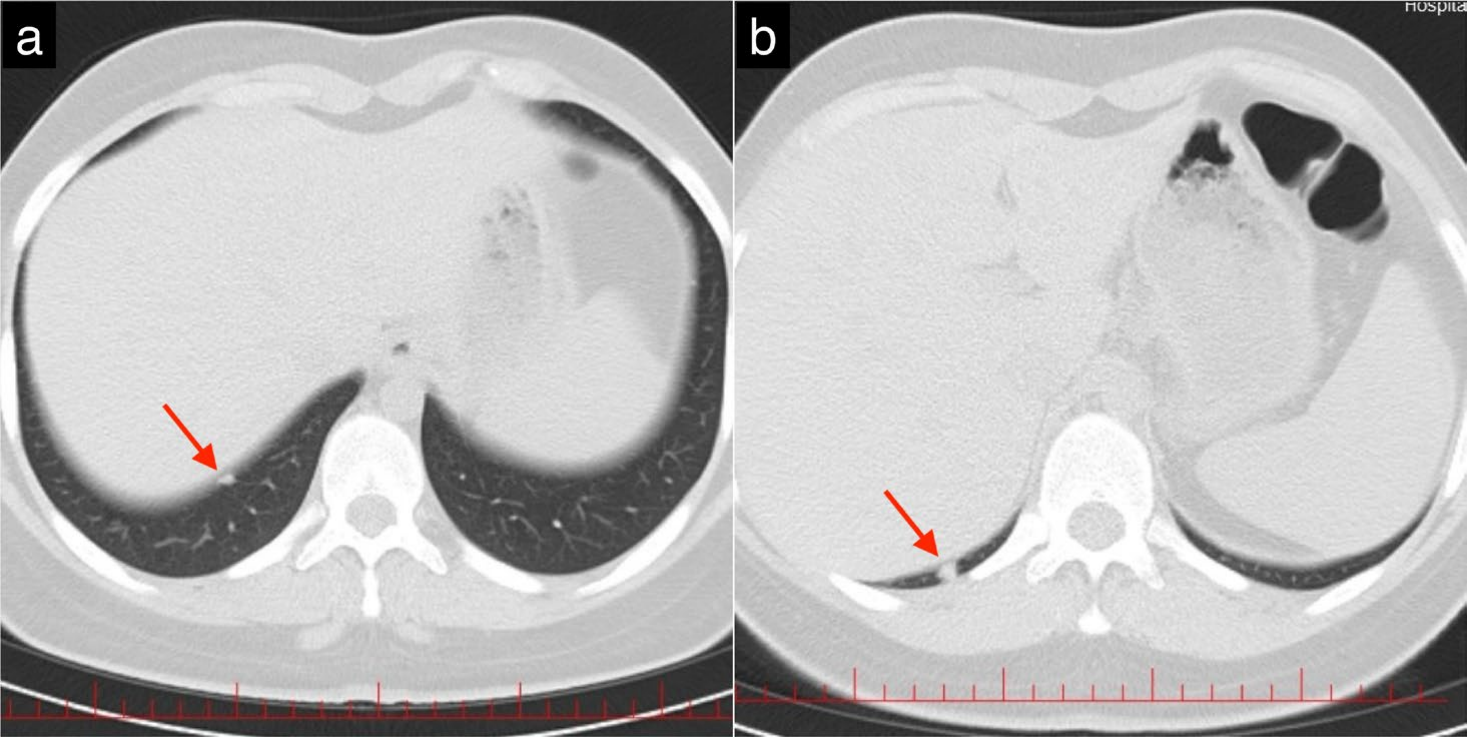
HRCT of lungs. (a and b) Well‐circumscribed parenchymal nodules (red arrows).

**FIGURE 4:**
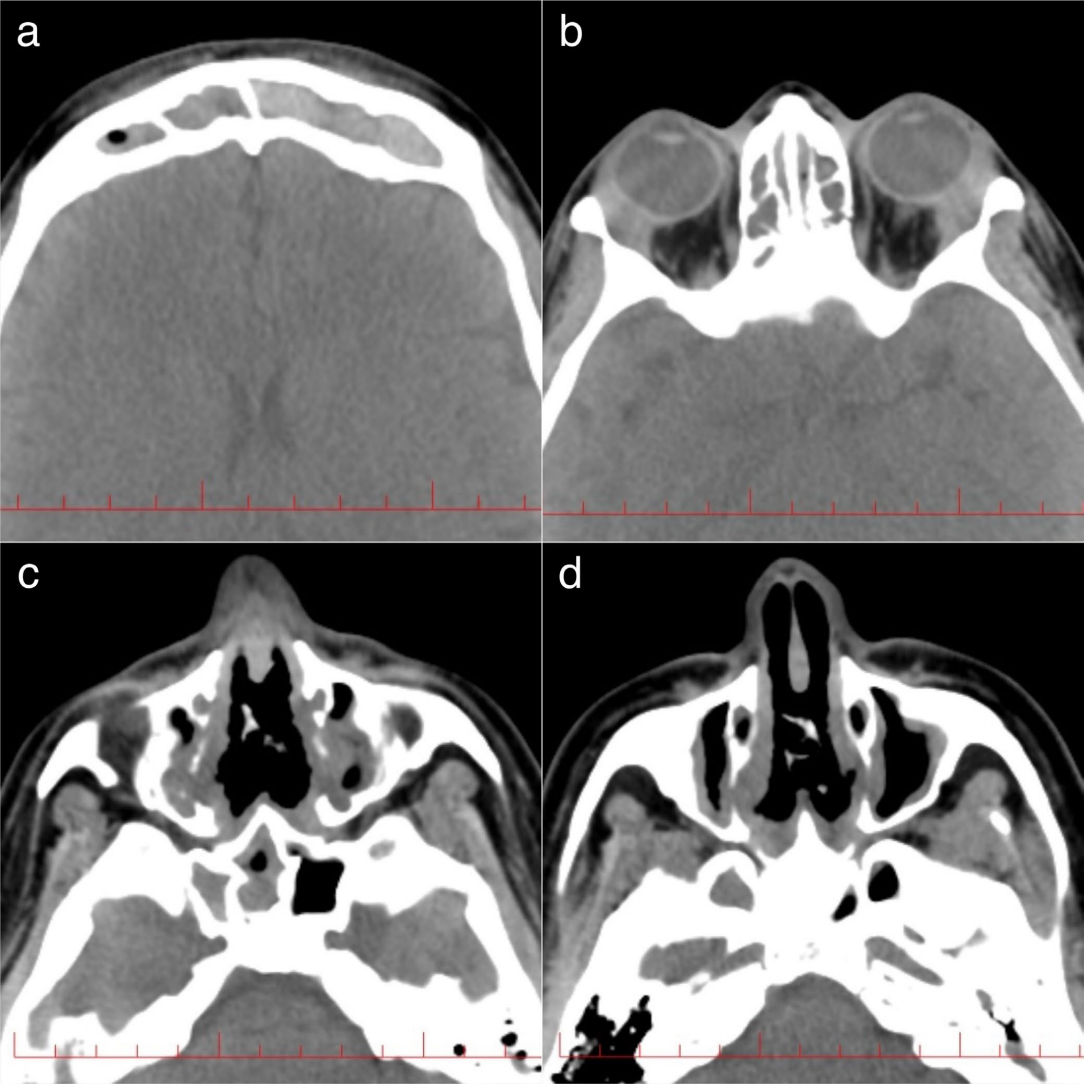
CT scan of paranasal sinuses indicating pansinusitis and nasal septum defect. (a) Frontal sinuses; (b) Ethmoidal sinuses; (c) Sphenoid and maxillary sinuses; (d) Nasal septum defect.

Considering the patient's neurological and ocular signs and symptoms, a brain and orbital MRI without contrast agent was ordered, which revealed enhancement of the pituitary stalk suggestive of hypophysitis (Figure 2). Due to the history of dyspnea, the patient underwent an electrocardiogram (ECG) and echocardiography, which were normal, and a high‐resolution computed tomography (HRCT) was performed, which revealed three well‐circumscribed parenchymal nodules with a maximum diameter of 6 mm, with no evidence of pleural effusion, lymphadenopathy, or pathological mediastinal lesions (Figure 3). Additionally, a CT scan of the paranasal sinuses was performed, which indicated severe mucosal thickening in all sinuses in favor of pansinusitis, along with a nasal septum defect (Figure 4). According to these findings and the seasonal nature of the patient's dyspnea, her dyspnea was probably due to sinus involvement.

**FIGURE 5:**
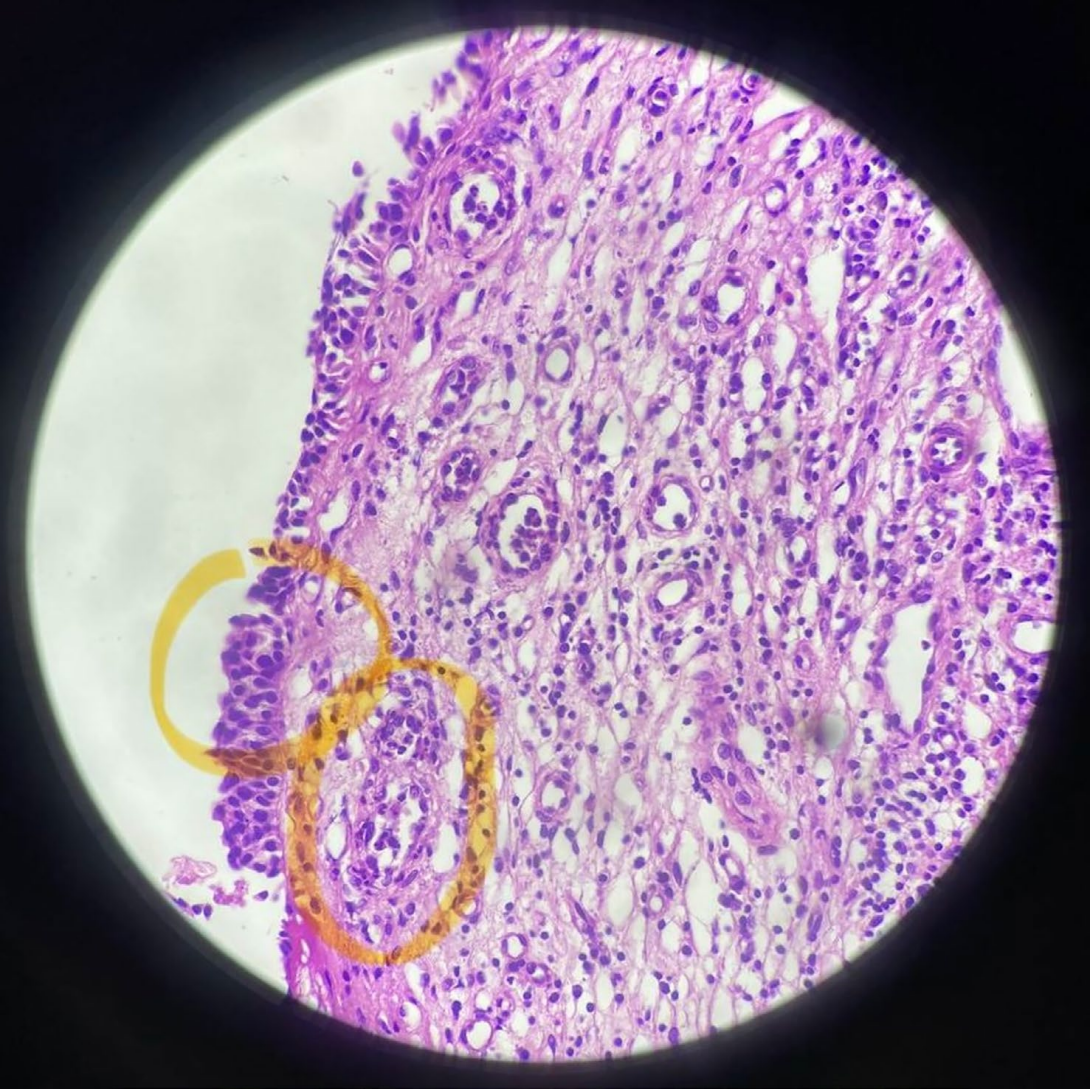
Histological finding of nasal mucosa biopsy showing small vessel vasculitis and fibrosis without granulomatous inflammation (yellow circles).

The differential diagnosis of hypophysitis is broad and includes a variety of autoimmune, infiltrative, infectious, and neoplastic processes, among which, due to the sinonasal involvement and respiratory symptoms of our patients, sarcoidosis, granulomatosis with polyangiitis, IgG4‐related diseases, and lymphoma were the most likely diagnoses. Additionally, pituitary tuberculoma, a rare presentation of tuberculosis, can be relevant, considering that the patient is from an endemic region. To address the differential diagnosis, a baseline laboratory investigation consisting of a complete blood count, erythrocyte sedimentation rate (ESR), C‐reactive protein (CRP), calcium, creatinine, urinalysis, and liver function tests, in addition to rheumatology biomarkers, antineutrophil cytoplasmic antibodies, IgG4, ACE, B2‐microglobulin, and tuberculin skin test were performed, which indicated high levels of rheumatoid factor (RF) followed by a normal range of anti‐CCP, and an increase in P‐ANCA levels, followed by a normal range of C‐ANCA. Additionally, increased levels of CRP (20.12 mg/L) and ESR (38 mm/h), in conjunction with mild anemia (Hb:11.3 g/dL and MCV: 74.2), were noted, but there were no other notable findings, and blood urea nitrogen (BUN), creatinine, electrolytes, liver function, and urinalysis tests were all within the normal range. The patient then underwent nasal endoscopy, in which severe nasal crusting was observed. After debridement, evidence of inflamed and bleeding nasal mucosa with a perforated nasal septum was noted. A nasal mucosa biopsy was subsequently taken from eight different sites. Later, the pathology report indicated small‐vessel vasculitis and fibrosis without granulomatous inflammation in the nasal mucosa sample (Figure 5).

On the basis of the patient's history of recurrent sinusitis and hearing loss, physical examination findings of the saddle nose and perforated nasal septum, imaging findings of pansinusitis and multiple lung nodules, high levels of P‐ANCA and RF in laboratory data, and finally, evidence of small vessel vasculitis in the pathology study, granulomatosis with polyangiitis (GPA) was the most likely diagnosis for the patient. According to this diagnosis, three pulses of intravenous methylprednisolone (1 g) were prescribed for 3 days, followed by prednisolone (60 mg daily), methotrexate (10 mg weekly), folic acid (1 mg daily), and calcium D. The patient was also given trimethoprim/sulfamethoxazole, a proton pump inhibitor (PPI), vitamin D, and a calcium supplement. Considering the age of the patient and possible infertility due to cyclophosphamide and consultation with the patient's family, she was given rituximab instead.

## 4 Conclusion and Results (Outcome and Follow‐Up)

Following the mentioned treatment, respiratory, neurological, and ocular involvement, including dyspnea, headache, and diplopia, improved. Finally, after 17 days of hospitalization, the patient was discharged from the internal ward in good general condition. She attended follow‐up visits after 2 and 4 weeks, during which time the patient's urinary and other symptoms and signs improved, and the prednisolone tapering process was started. Other medications were continued, as mentioned previously. At the time of submission of this report (approximately one year after initiation of treatment), the patient had no recurrence of symptoms.

## 5 Discussion

Granulomatosis with polyangiitis, previously known as Wegener's granulomatosis, is an autoimmune disease characterized by necrotizing pauci‐immune vasculitis affecting small‐to‐medium vessels that can impact various organ systems [10]. The most affected organs are the upper and lower respiratory tract, lungs, and kidneys [11]. Nevertheless, GPA can potentially involve any organ in the body, including the skin, eyes, trachea, central and peripheral nervous systems, gingiva, breast, and prostate [12]. Our patient also had different organ involvement; as a result, she experienced dyspnea, epistaxis, gingiva bleeding, and recurrent sinusitis. CNS involvement occurs in 7%–11% of patients, in which common central nervous system manifestations are cranial neuropathies, cerebral vasculitis, and meningitis following the granulomatous process from the sinuses, orbits, and vasculitis of the cerebral vessels [13, 14]. Pituitary dysfunction (PD), which was a major manifestation in our patient, is an uncommon presentation with an incidence of approximately 2% and tends to manifest during the progression of the disease [3]. The precise way in which the pituitary is affected remains uncertain. Three different mechanisms are recommended to explain pituitary involvement in GPA: vasculitis of the pituitary vessels leading to ischemia and hemorrhagic infarctions; the development of granulomas in the pituitary gland; and direct granulomatous extension from adjacent anatomical areas such as the ear, nose, and throat [12]. Inflammatory and granulomatous lesions in the pituitary gland are rare causes of PD [15, 16]. Hypophysitis refers to acute or chronic inflammation of the pituitary gland and can be categorized as adenohypophysitis, infundibulohypophysitis, or panhypophysitis, depending on the site of involvement [15]. It is shown that 13% of cases had only anterior pituitary abnormalities, 52% had only central diabetes insipidus, and 35% had both anterior and posterior pituitary involvement [17]. Diabetes insipidus is the most common presentation of Wegener's granulomatosis with PD, as observed in our patient. However, a few case reports have described Wegener's granulomatosis involving the anterior pituitary gland, resulting in hyperprolactinemia or panhypopituitarism [18, 19, 20].

While clinical features offer valuable diagnostic clues, serological investigations such as testing for antineutrophil cytoplasmic antibodies, are crucial in confirming and supporting the diagnosis. Two major ANCA types are directed against proteinase 3 (PR3) and myeloperoxidase (MPO). PR3‐ANCA (C‐ANCA) is associated mainly with GPA, and MPO‐ANCA (P‐ANCA) is associated with microscopic polyangiitis (MPA); however, as seen in our case, discordant ANCA types, such as P‐ANCA‐positive GPA, are sometimes observed in certain populations, particularly in Asia [21]. Radiological assessments indicate pituitary enlargement in most cases [22]. While MRI findings in patients with GPA‐induced hypopituitarism are well‐characterized, they may not always correlate directly with disease progression or resolution. The most common finding is pituitary enlargement or adenoma, diffuse or focal infundibular thickening, and the absence of posterior hypersignal on T1‐weighted images, described in 80% of patients [10, 23]. If the diagnosis remains uncertain or needs confirmation, a biopsy can provide valuable information, as a nasal mucosa biopsy was performed in our patient, confirming the diagnosis of GPA [8, 10, 24].

GPA treatment relies mainly on various immunosuppressing agents for remission induction and maintenance, such as glucocorticoids and cyclophosphamide [7]. The combination of cyclophosphamide and high‐dose glucocorticoids has been used most frequently, and historically, this approach has significantly reduced 1‐year mortality rates from 80% to 10%–20% [25]. Recent studies have shown that rituximab (RTX) is a suitable alternative to cyclophosphamide for remission induction, as it was preferred in our case because of the young age of the patient and the possibility of infertility associated with cyclophosphamide [26]. After receiving immunosuppressive treatment, enlarged pituitary glands may decrease in size [27]. Conventional remission‐induction treatment for PD in GPA patients consists of high‐dose glucocorticoids combined with oral or intravenous cyclophosphamide, with reported remission in two‐thirds of patients [28]. Nevertheless, for some patients with progressive disease, pituitary resection may be necessary to alleviate the effects of granulomatosis. Our patient received combination therapies, including high‐dose glucocorticoids with immunosuppressant agents (rituximab). RTX treatment was generally well tolerated, and our patient reported neither significant infections nor side effects. Approximately 67%–88% of patients treated with immunosuppressive agents will experience GPA remission. In patients whose pituitary function is restored, the resolution of diabetes insipidus occurs significantly more often than the return of adenohypophysis function, which is infrequent and may indicate more extensive disease [10].

Considering the nature of vasculitic diseases, which potentially involve many organs and, as a result, each patient can be different from another, it is very important to consider such diseases when evaluating patients with various types of involvement. Then, based on clinical suspicion, comprehensive evaluations, as well as targeted laboratory and paraclinical studies, should be performed to reach a correct diagnosis. Due to the low prevalence of central nervous system involvement in GPA, we present a rare case of pituitary dysfunction with central diabetes insipidus manifestation and cranial nerve involvement. It is important to consider clinical judgment as well as laboratory data and imaging findings to lead to a correct diagnosis of Wegener's granulomatosis of the pituitary gland. Given the awareness of the potential involvement of the pituitary gland in GPA, unnecessary biopsies or surgical procedures could be prevented. A combination of glucocorticoids and RTX can be used to treat systemic GPA complicated by pituitary gland involvement. The follow‐up of patients with CNS manifestations of this disease is critical and should include imaging and pituitary function tests.

## Author Contributions

**Mina Kiafar:** supervision. **Goharsharieh Alishiri:** writing – original draft, writing – review and editing. **Ahmadreza Sadeghi:** writing – original draft, writing – review and editing. **Amirreza Paksaz:** writing – original draft. **Leila Khezrloo:** writing – review and editing. **Soheila Borji:** supervision.

## Ethics Statement

Ethics approval for this study was provided by the Ethical Committee of Imam Ali Hospital, Karaj, Iran. This study complies with ethical and research standards involving humans.

## Consent

Written informed consent was obtained from the patient for publication and any accompanying images. A copy of the written consent is available for review by the Editor‐in‐Chief of this journal upon request.

## Conflicts of Interest

The authors declare no conflicts of interest.

## Data Availability

The data that support the findings of this study are available on request from the corresponding author. The data are not publicly available due to privacy or ethical restrictions.
